# Impact of Nordic hamstring breaking point angle on football player performance

**DOI:** 10.7717/peerj.19275

**Published:** 2025-04-23

**Authors:** Murat Koç, Niyazi Sıdkı Adıgüzel, Barışcan Öztürk, Hakan Engin, Aydın Karaçam, Umut Canli, Bekir Erhan Orhan, Pablo Prieto-González, Peter Sagat, Jason Perez, Maria Isip, Peter Bartik

**Affiliations:** 1 Faculty of Sport Sciences, Erciyes University, Kayseri, Türkiye; 2 Faculty of Sport Sciences, Bandırma Onyedi Eylül University, Balıkesir, Türkiye; 3 Faculty of Sport Sciences, Çukurova University, Adana, Türkiye; 4 Faculty of Sport Sciences, Tekirdağ Namık Kemal University, Tekirdağ, Türkiye; 5 Faculty of Sport Sciences, İstanbul Aydın University, İstanbul, Türkiye; 6 Sport Sciences and Diagnostics Research Group, College of Humanities and Sciences, Prince Sultan University, Riyadh, Saudi Arabia; 7 Preparatory Year Program, College of Humanities and Sciences, Prince Sultan University, Riyadh, Saudi Arabia

**Keywords:** Hamstring muscle strength, Performance in football players, Change of direction, Nordic Hamstring breaking point angle

## Abstract

**Background:**

Football demands both aerobic and anaerobic capacities due to its dynamic movements, which include jumps, directional changes, ball control, and sprints. The Nordic hamstring exercise (NHE) enhances eccentric strength, which is crucial for high-intensity movements. However, the relationship between Nordic breaking point angle (NHEbpa), which is associated with eccentric hamstring peak torque during Nordic hamstring exercise, and sprint, countermovement jump (CMJ), and change of direction (COD) speed in soccer players has not been sufficiently investigated.

**Objective:**

This analysis examines the relationship between the Nordic breaking point angle and critical performance indicators—sprint speed, COD ability, and vertical jump—in football players.

**Methods:**

Fifty-eight male soccer players volunteered for the study. Assessments included anthropometric measurements, CMJ tests, 10-20-30 m sprint tests, and COD (zig-zag) tests. NHEbpa was measured using motion analysis software. Correlation analysis was used to determine the relationship between variables. A multiple linear regression analysis was conducted to evaluate the individual effects of the sprint, CMJ, and COD performances on NHEbpa, with data analyzed using JASP 0.18.

**Results:**

Correlation analysis showed strong positive correlations between NHEbpa and sprint performances (r = 0.633 to 0.666), moderate negative correlation between NHEbpa and CMJ (r = −0.406), and moderate positive correlation between NHEbpa and COD (r = 0.580). Regression analysis results were used to analyze the independent coefficients of multiple variables more comprehensively, revealing significant predictors for performance: 20-m sprint (β = 24.166, *p* = 0.030), 10-m sprint (β = 22.564, *p* = 0.047), 30-m sprint (β = 10.677, *p* = 0.027), and CMJ (β = 4.974, *p* = 0.034). Conversely, COD performance (β = −0.154, *p* = 0.470) did not demonstrate a significant effect.

**Conclusions:**

The study identified significant relationships between NHEbpa and sprint/CMJ performances (*p* < 0.05), while no meaningful effect was observed for COD speed (*p* > 0.05). These findings highlight the importance of eccentric strength in sprint performance, suggesting that other factors may play a more prominent role in COD. Incorporating eccentric-focused training, particularly through Nordic exercises, is recommended to enhance sprint performance and hamstring strength, which are essential for football players.

## Introduction

Football is an athletic pursuit characterized by dynamic movements such as jumps, changes of direction, ball control, and sprints, involving a combination of aerobic and anaerobic capacities, where technical expertise and physical and tactical skills are required to achieve peak performance (Rouissi et al., 2016; Teixeira et al., 2022). As a result, an athlete’s football performance must be evaluated from a multifaceted perspective (Kelly et al., 2022).

The Nordic hamstring exercise (NHE) is an exercise that involves eccentric contractions that mainly aim to strengthen the hamstring muscles (Lee et al., 2017; Ripley et al., 2023). The primary purpose of NHE is to increase eccentric hamstring strength, which supports high-intensity movements such as sprinting and change of direction (COD). Eccentric training offers several advantages over traditional resistance exercise, including producing supra-maximal force with lower energy expenditure. Research in this area has shown that eccentric contractions increase muscle strength and improve neuromuscular activation, contributing to overall health and athletic performance (Hody et al., 2019; Paschalis et al., 2013).

Eccentric exercises have long been a cornerstone of strength training and rehabilitation programs, particularly in preventing injuries such as hamstring strains (Ayala et al., 2024; Croisier et al., 2007; Kaux et al., 2013). Recent studies have focused on low- and moderate-load eccentric training regimens, highlighting their effectiveness in enhancing performance and preventing injuries (Burgos-Jara et al., 2023; Cvečka et al., 2023; Hoppeler, 2014; LaStayo et al., 2014). Specifically, it has been demonstrated that the NHE significantly enhances eccentric hamstring strength, which is essential for rapid acceleration and deceleration movements such as sprinting and COD (Ishoi et al., 2018; Mendiguchia et al., 2015; Moran et al., 2022). In this context, the Nordic breaking point angle (NHEbpa) emerges as a fundamental biomechanical parameter reflecting the maximum tension the hamstring muscles can tolerate during eccentric contractions, such as the NHE, and its relationship to eccentric hamstring peak torque (Lee et al., 2017). This angle represents the maximum tension the hamstring muscles can withstand during eccentric contractions (Sconce et al., 2015; Sconce et al., 2021). Furthermore, technological advancements have made NHEbpa more accessible (Soga et al., 2023). One study highlighted that NHEbpa was significantly correlated with eccentric hamstring peak torque and may serve as an indirect measure of hamstring power capacity (Lee et al., 2017). This correlation suggests that monitoring NHEbpa may provide valuable information regarding an athlete’s eccentric strength, which is important for performance and injury prevention. Because hamstring strength at the breakdown point can impact performance by increasing force production during high-intensity movements, improving sprint mechanics, and supporting neuromuscular control during direction changes (Jiang et al., 2023; Váczi et al., 2022). While the relationship between eccentric hamstring strength and performance measures such as sprinting and COD has been investigated, the impact of the NHEbpa—the angle at which the athlete can no longer withstand the eccentric load—on key performance indicators such as sprint times, COD, and vertical jump performance has not been adequately studied. Because hamstring strength at the breakdown point can impact performance by increasing force production during high-intensity movements, improving sprint mechanics, and supporting neuromuscular control during direction changes (Jiang et al., 2023; Váczi et al., 2022).

This analysis examines the relationship between NHEbpa and important performance measures such as sprint speed, CMJ, and COD in football players. An increase in NHEbpa indicates a more significant deviation toward knee extension during the Nordic hamstring exercise, which may reduce joint stability and neuromuscular control. This could negatively impact force transmission efficiency in sprinting and jumping movements and potentially increase the risk of injury (Enomoto, Oda & Kaga, 2019; Kubo et al., 2007). By analyzing these relationships using motion analysis software, we intend to provide insights into the practical applications of NHEbpa for optimizing performance in football.

## Materials and Methods

### Research design

**Figure 1:**
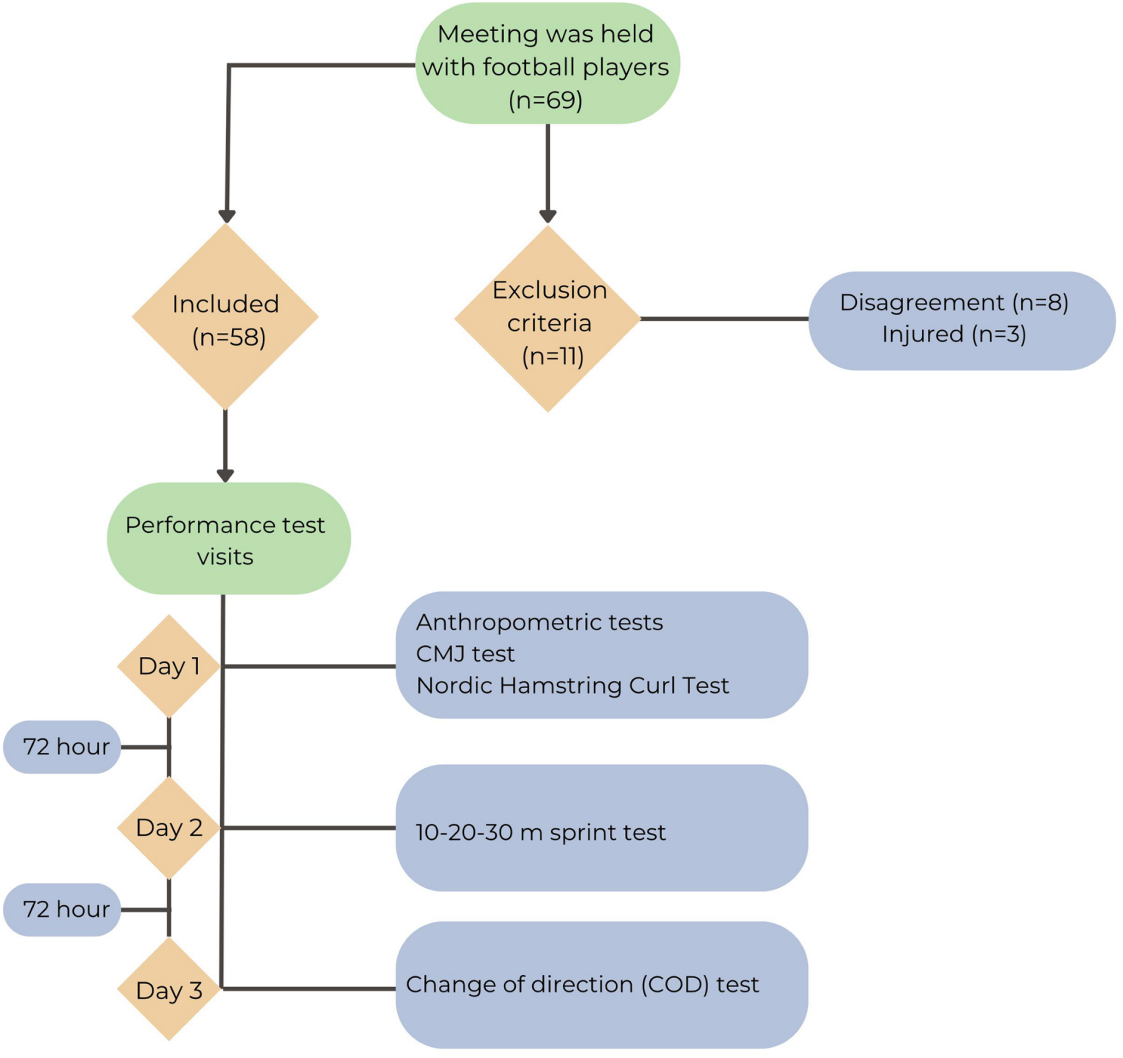
Research design.

The study included several performance tests: anthropometric measurements, CMJ, NHEbpa, and sprint tests over distances of 10, 20, and 30 m, as well as a COD test. It was conducted over three sessions, each separated by a 72-h interval, to facilitate recovery and minimize the potential effects of fatigue or learning. In the first session, participants underwent anthropometric measurements, the CMJ test, and the NHEbpa assessment. The second session focused on the sprint tests (10, 20, and 30 m), while the final session was dedicated to the COD test. Before each test session, the athletes performed a conveniently standardized warm-up divided into phases: 5 min of aerobic exercise, which included jogging, running and mobility exercises such as skipping and kicking backwards; a 5-min neuromuscular phase that included strength exercises such as squat and balance exercises, and finally a 5 min phase of linear and non-linear sprint bouts (Ben Brahim et al., 2023). All tests were conducted at the semi-professional Adana Vefa Sports Club facilities, with the CMJ and NHEbpa assessments in the athletic performance studio. Conversely, the sprint and COD tests were performed on the team’s official grass field under average conditions of 29 °C temperature, 37% humidity, and barometric pressure ranging from 1,010 to 1,025 mmHg. The research was conducted between March and June, towards the end of the season. To mitigate the effects of circadian rhythm on the athletes, the same researcher conducted the study at consistent times each day (17:00–18:00) (Adıgüzel et al., 2024). The study participants were verbally encouraged to perform to the best of their ability (Pacholek & Zemková, 2022). In order to ensure consistency in management and reduce possible measurement variability, all 58 participants completed all the tests in the specified order (Fig. 1). The institutional review board approved the protocol of Çukurova University’s ethical committee (approval number: 2024-143), and all procedures adhered to the principles of the Declaration of Helsinki. After detailed information about the study was given to all participants and their legal heirs, written consent was obtained from all participants. The anonymization of personal data ensured participant confidentiality. All collected information was kept confidential and securely stored.

### Sample size

The study’s sample size was calculated using the G*Power program (version 3.1.9.2), with an effect size of f^2^ = 0.15, α = 0.05, and power = 0.80 (1-β) (Cohen, 2013; Kang, 2021). Based on the Exact analysis group, the required number of participants for linear regression analysis was determined to be *n* = 43. To enhance the validity of the research and minimize potential data loss, the study ultimately included 58 participants.

### Participants

A total of 58 male football players participated in the research. The athletes had an average sports experience of 5.68 ± 1.63 years, an age of 16.84 ± 0.61 years, a height of 1.77 ± 0.05 m, a body mass of 67.48 ± 5.51 kg, and a body mass index (BMI) of 21.47 ± 1.72 kg/m^2^

Inclusion Criteria: Male soccer players aged 16 to 18 with at least 3 years of playing experience. Participants had no current injuries at the time of the study.

Exclusion Criteria: Participants with a history of injuries requiring medical treatment or rehabilitation within the past year were excluded. Additionally, those with chronic conditions affecting physical performance or a history of injuries to the tested muscle groups (hamstrings, quadriceps, calves) were excluded from the study. Injury history was assessed using a detailed questionnaire administered before participation.

This meticulous approach ensured that the sample consisted of healthy, active football players representative of the target population, allowing for a clearer understanding of the relationship between performance metrics and the NHEbpa.

### Data collection

#### Body mass and height measurement

Athletes’ height (measured with a sensitivity of 0.5 cm) and body mass (measured with a sensitivity of 100 g) were assessed using a Seca stadiometer. The researchers also collected demographic characteristics, including age and sports age, through a questionnaire.

#### Counter movement jump (CMJ)

The CMJ tests for athletes were measured using a Witty Microgate jumping mat (Multhuaptff et al., 2023). Participants jumped to the highest point at the midpoint of the jump mat while keeping their hands free. The height of each jump was recorded, and each athlete completed three attempts. The average jump height was calculated for analysis (Glatthorn et al., 2011). This calculation method may not fully account for possible differences in jump technique or landing mechanisms, which may affect the accuracy of flight time (McMahon, Jones & Comfort, 2016).

#### 10-20-30 m sprint test

The 10-20-30 m sprint test was conducted using the Witty Microgate photocell system along a 30 m track (synthetic grass) (Multhuaptff et al., 2023). Photocells were positioned 10, 20 m, and 30 m from the starting line. Each athlete started from 50 cm behind the photocell gate to trigger the digital timer. Each athlete performed two maximal sprints with at least 2 min of passive recovery between attempts. The best performance was recorded for analysis (Fernandez-Fernandez et al., 2018).

#### Change of direction test (COD-Zigzag test)

The COD test was conducted using the Witty Microgate photocell system (Multhuaptff et al., 2023). This test consists of three slaloms arranged in a zig-zag pattern, spaced 5 m apart, with an angle of 100 degrees between each slalom over a total distance of 20 m (synthetic grass). Athletes initiated the test 1 m behind the starting line and navigated through the slaloms at maximum speed. Each athlete completed two trials, and the best time recorded was used for analysis (Loturco et al., 2016; Pereira et al., 2018).

#### Nordic hamstring curl breaking point angle

Participants knelt on a mat with their elbows bent and hands open in front of them while a teammate held their feet steady behind them. Athletes were instructed to lean forward slowly, maintaining a straight posture from knee to head. A researcher ensured the correct execution of the NHE. The movement of the NHEbpa was determined through 2D motion analysis. An iPhone 14 camera was set to 240 fps and positioned approximately 3 m to the participants’ right at a height of around 0.9 m. After transferring the recorded video to a personal computer, two-dimensional motion analysis was conducted using specialized software (KİNOVEA Inc.). This analysis determined the angle from the knee to the ground when the athlete lost balance and stopped the NHE movement, defining it as the breaking point angle (NHEbpa) (Sconce et al., 2015; Soga et al., 2023). The measurement was repeated three times, and the best score was recorded.

### Statistical analysis

Statistical analyses were conducted using JASP version 0.18. The normality of the data was initially evaluated through skewness and kurtosis tests. Normality was assumed if the values fell between −1.5 and +1.5, following the guidelines established by Kim (2013). The results indicated that the data followed a normal distribution. Pearson correlation coefficients were calculated to examine the relationships between the NHEbpa and the football players’ sprint, COD, and CMJ performances. Due to the observed multicollinearity, Ridge Regression was employed to address this issue. Ridge Regression was preferred over alternative methods, such as stepwise regression, as it is particularly effective in cases of high multicollinearity. While stepwise regression selects variables by iteratively including or excluding them, Ridge Regression does not eliminate variables but instead adds a penalty term to the regression equation. This penalty term shrinks the regression coefficients, stabilizing them and thereby improving the reliability and interpretability of the model. In cases of high multicollinearity, Ridge Regression allows for more stable and accurate predictions by reducing the influence of correlated independent variables (Efron et al., 2004). Pearson correlation strengths were interpreted according to established definitions: an R-value from 0.00 to 0.19 was deemed “very weak,” 0.20 to 0.39 as “weak,” 0.40 to 0.59 as “moderate,” 0.60 to 0.79 as “strong,” and 0.80 to 1.0 as “very strong” (Evans, Heath & Lalljee, 1996). The significance level for all statistical tests was set at *p* < 0.05. The reliability of the measurements was assessed using the intraclass correlation coefficient (ICC), which ranges from 0 to 1. Reliability levels were categorized based on the 95% lower bound confidence intervals as follows: 0.00 to 0.49 as “poor,” 0.50 to 0.75 as “moderate,” 0.75 to 0.90 as “good,” and 0.90 to 1.00 as “excellent” (Koo & Li, 2016). A multiple linear regression analysis was initially conducted to evaluate the individual effects of the sprint, CMJ, and COD performances on NHEbpa. However, due to significant multicollinearity among the predictors, Ridge Regression was employed to enhance the analysis and mitigate the effects of multicollinearity (Efron et al., 2004). The significance level was maintained at *p* < 0.05 throughout the analysis.

## Results

A total of 58 male football players participated in the research (Table 1). When the NHEbpa, sprint, COD, and CMJ performances of football players were examined, the following results were obtained: NHEbpa angle was 45.08 ± 7.73 degrees (95% CI [43.05–47.11]), CMJ was 1.74 ± 0.07 cm (95% CI [0.607–0.862]), 10-m sprint was 3.08 ± 0.08 s (95% CI [1.72–1.77]), 20-m sprint was 4.26 ± 0.17 s (95% CI [3.06–3.11]), 30-m sprint was 5.29 ± 0.34 s (95% CI [4.21–4.30]), and COD performance was 36.87 ± 3.52 s (95% CI [35.95–37.80]). The intraclass correlation coefficients (ICC) for these measures were high, with 0.92 for NHEbpa, 0.82 for CMJ, 0.77 for a 10-m sprint, 0.83 for a 20-m sprint, 0.82 for 30-m sprint, and 0.92 for COD, indicating strong reliability for these performance tests (Table 2).

**Table 1:** Demographic characteristics.

	*n*	Min.	Max.	${\mathrm{\bar x}}$	Std.
Age (year)	58	16.00	18.00	16.84	0.61
Height (m)	58	1.65	1.90	1.77	0.05
Body mass (kg)	58	60.00	77.00	67.48	5.51
BMI (kg/m^2^)	58	17.86	25.06	21.47	1.72
Training age (year)	58	2.00	10.00	5.68	1.63

**Table 2:** Descriptive statistics and reliability measures for NHCEbpa, Sprint, COD, and CMJ scores.

Variable	${\mathrm{\bar x}}$ ± Std.	95% CI	ICC (95% CI)
NHEbpa^o^	45.08 ± 7.73	[43.05–47.11]	[0.85–0.96]
10 m (s)	1.75 ± 0.08	[1.72–1.77]	[0.68–0.87]
20 m (s)	3.08 ± 0.17	[3.06–3.11]	[0.76–0.91]
30 m (s)	4.26 ± 0.34	[4.21–4.30]	[0.74–0.90]
CMJ (cm)	36.87 ± 3.52	[35.95–37.80]	[0.69–0.92]
COD (s)	5.29 ± 0.34	[5.20–5.39]	[0.85–0.96]

**Note:**

NHEbpa^o^, Nordic Hamstring Breaking Point Angle; CMJ, Counter Movement Jump; COD, Change of Direction; ICC, Intraclass Correlation Coefficient; 95% CI, 95% Confidence Interval.

Strong positive correlations were found between NHEbpa and sprint performances (10: r = 0.633, 20 m: r = 0.666, 30 m: r = 0.607; *p* < 0.001). A moderate negative correlation was found between NHEbpa and CMJ (r = −0.406, *p* = 0.002). A moderate positive correlation was observed between NHEbpa and COD (r = 0.580, *p* < 0.001) (Table 3).

**Table 3:** Correlations between nordic hamstring curl break point and sprint, change of direction and jump performance.

	*n*		10 m	20 m	30 m	CMJ	COD
NHEbpa°	58	r	0.633	0.666	0.607	−0.406	0.580
		*p*	<0.001	<0.001	<0.001	0.002	<0.001

**Note:**

NHEbpa°, Nordic Hamstring Breaking Point Angle; CMJ, Counter Movement Jump; COD, Change of Direction.

The relationship between the dependent variable, the NHEbpa, and the independent variables—including the COD test, CMJ, 20-m sprint, 10-m sprint, and 30-m sprint—explains 62.9% of the variance in the NHEbpa (R^2^ = 0.629, Adjusted R^2^ = 0.593). The Durbin-Watson statistic was calculated as 1.067, indicating no significant autocorrelation in the residuals (Table 4).

**Table 4:** Summary of regression model for predicting NHEbpa.

Model	R	R-squared	Adjusted R-squared	Standard error	R-squared change	F	*p*
	0.793	0.629	0.593	4.933	0.629	17.622	*p* < 0.001

**Note:**

Durbin watson: 1.065.

**Figure 2:**
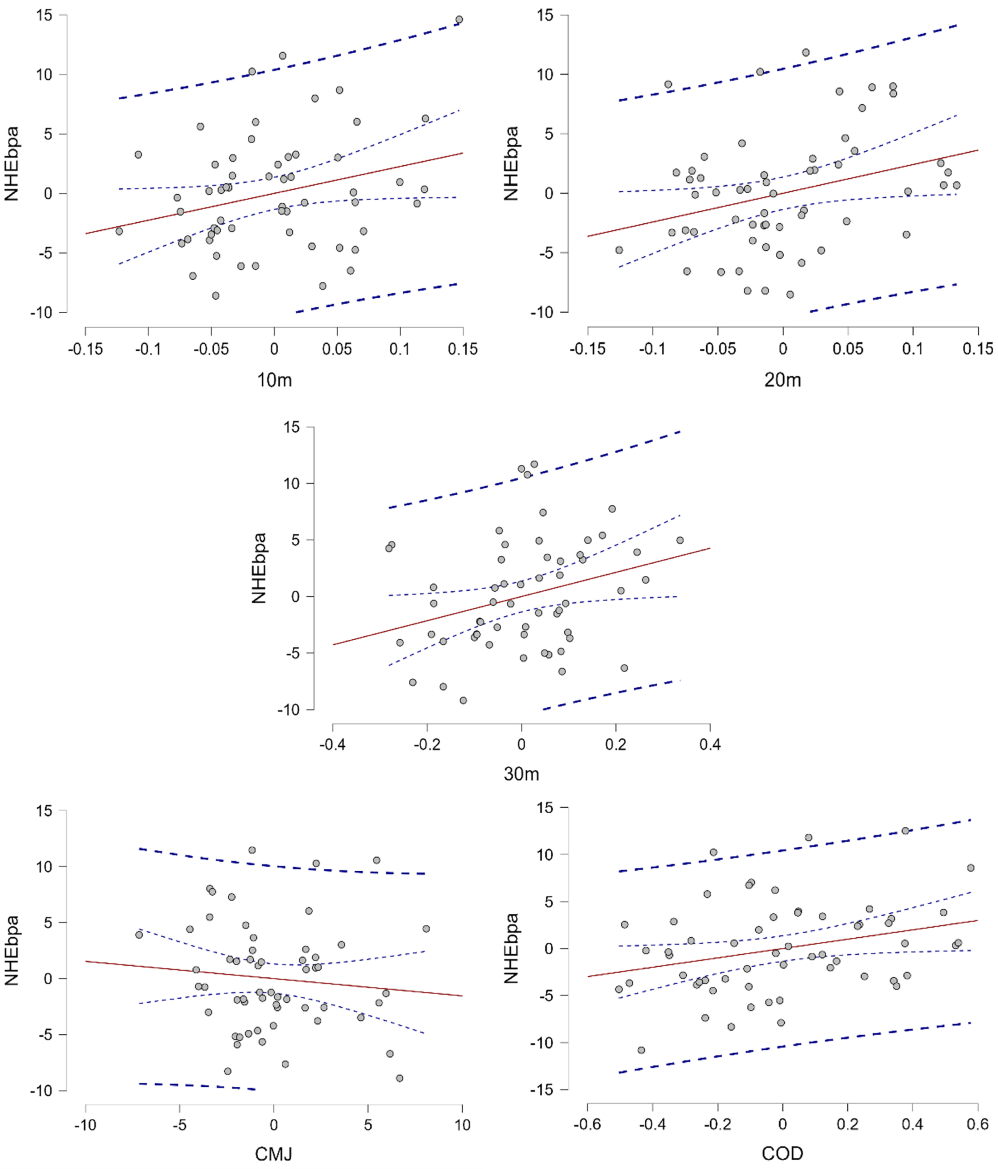
Residual plot showing the relationship between residuals of the NHEbpa and residuals of 10-m, 20-m, 30-m sprint, COD, and CMJ performance. The red line represents the linear trend of the residuals, and the dashed blue lines represent confidence intervals or limits of variability.

The regression analysis results showed that the 20-m sprint time significantly affected the NHEbpa, with a 1-unit increase to 24.166 units (17.89%, *p* = 0.030). This was followed by the 10-m sprint time, where a 1-unit increase led to 22.564 units (16.71%, *p* = 0.047). The 30-m sprint time was also significantly affected, with a 1-unit increase to 10.677 units (7.91%, *p* = 0.027). Additionally, COD performance also had a significant effect on the NHEbpa. A 1-unit increase in COD performance (β = 4.974, *p* = 0.034) resulted in a significant increase of 4.974 units (3.68%) in NHEbpa. However, CMJ performance (β = −0.154, *p* = 0.470) did not have a significant effect on the NHEbpa (Table 5, Fig. 2).

**Table 5:** Coefficients of the regression model for the effect of sprint, COD, and CMJ performance on NHEbpa.

Model	β	Standard error	Beta	t	*p*	Tolerance	VIF	Correlations
(Constant)	−135,057	30,664		−4,404	<0.001			
10 m (s)	22,564	11,072	0.233	2,038	0.047	0.545	1,835	0.633
20 m (s)	24,166	10,798	0.266	2,238	0.030	0.504	1,985	0.666
30 m (s)	10,677	4,692	0.243	2,276	0.027	0.626	1,598	0.607
CMJ (cm)	−0.154	0.211	−0.070	−0.729	0.470	0.770	1,299	−0.406
COD (s)	4,974	2,290	0.221	2,172	0.034	0.692	1,446	0.580

**Note:**

NHEbpa, Nordic Hamstring Breaking Point Angle; CMJ, Counter Movement Jump; COD, Change of Direction.

Ridge Regression was employed to address multicollinearity among the predictors, and the results indicated that while an increase in CMJ negatively impacts the NHEbpa, higher scores in the 10, 20, and 30-m sprints, as well as in COD, positively affect the NHEbpa. Ridge Regression provided more stable and reliable estimates by mitigating the impact of multicollinearity, resulting in more accurate interpretations of the relationships between these variables and the NHEbpa (Table 5, Fig. 2).

**Figure 3:**
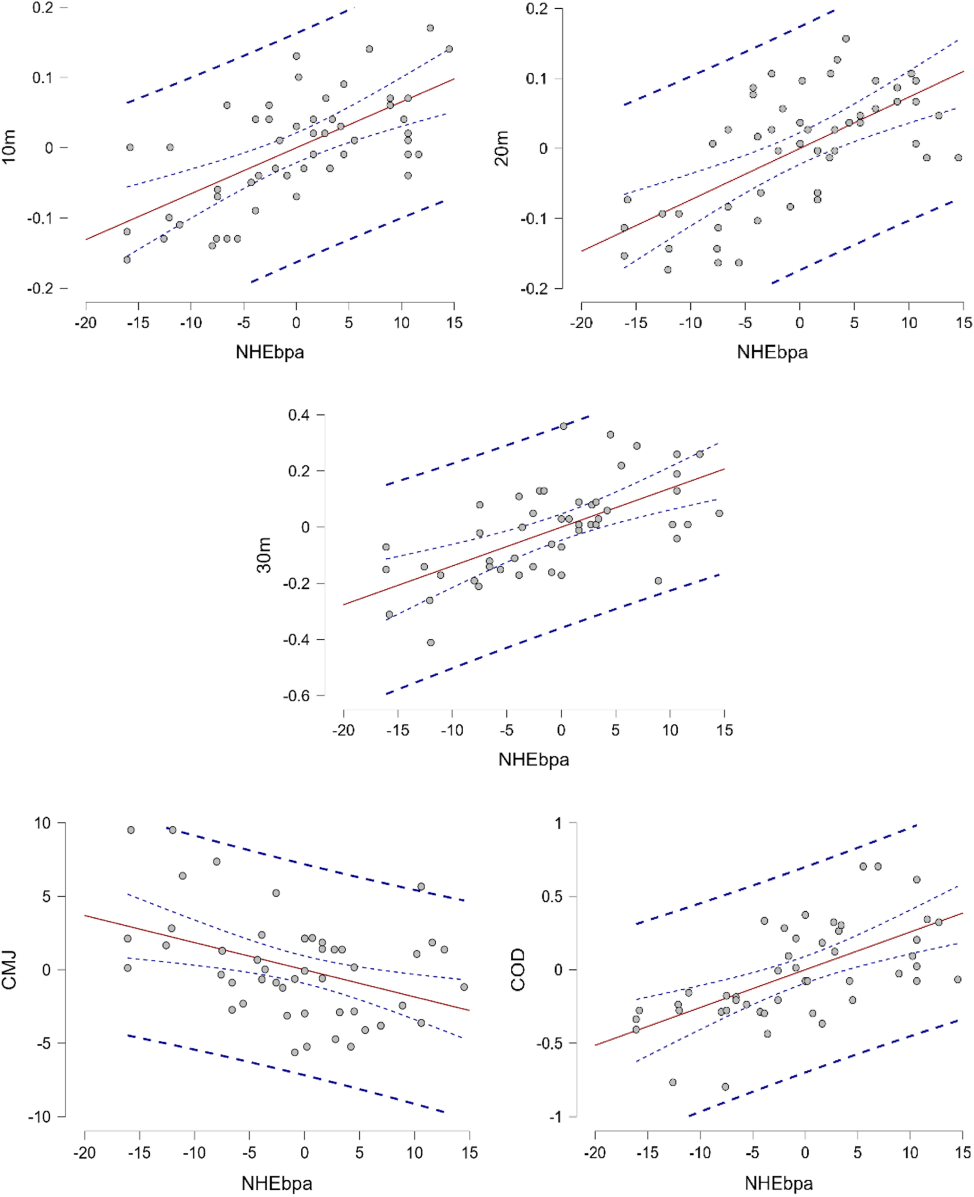
Residual graph showing the relationship between 10 m, 20 m, 30 m sprint, COD, and CMJ performance residuals and the NHEbpa. The red line represents the linear trend of the residuals, and the dashed blue lines represent confidence intervals or limits of variability.

The analysis demonstrates the significant effects of the NHEbpa on various performance metrics. For sprint performance, a robust positive relationship was identified: in the 10-m sprint, the NHEbpa explained 12.6% of the variance (β = 0.007, *p* < 0.001); for the 20-m sprint, this effect increased to 43.4% (β = 0.007, *p* < 0.001); and for the 30-m sprint, it accounted for 35.7% of the variance (β = 0.014, *p* < 0.001). Regarding jump performance, a higher NHEbpa was associated with reduced CMJ, accounting for 15% of the variance (β = −0.185, *p* = 0.002). For COD performance, the NHEbpa explained 32.5% of the variance (β = 0.026, *p* < 0.001). Overall, the NHEbpa significantly impacts sprint times, COD, and CMJ, with the most pronounced effects observed in the 20-m sprint (Table 6, Fig. 3).

**Table 6:** The effect of nordic hamstring curl breaking point on sprint, change of direction, and jump performance.

	β	Standart eror	Beta	t	p	r	r^2^	Durbin-Watson
10 m (s)	0.007	0.001	0.633	6,112	<0.001	0,355	0,126	1,845
20 m (s)	0.007	0.001	0.666	6,683	<0.001	0,444	0,434	1,821
30 m (s)	0.014	0.002	0.581	5,345	<0.001	0,368	0,357	1,481
CMJ (cm)	−0.185	0.056	−0.406	−3,321	0.002	0,165	0,150	1,577
COD (s)	0.026	0.005	0.580	5,332	<0.001	0,337	0,325	1,347

**Note:**

NHEbpa, Nordic Hamstring Breaking Point Angle; CMJ, Counter Movement Jump; COD, Change of Direction.

## Discussion

Previous studies on the Nordic Hamstring Curl exercise have demonstrated a gradual decrease in biceps femoris (BF) electromyography (EMG) activity following the break-point angle during the NHE (Sconce et al., 2021; Krommes et al., 2021; Murakami et al., 2023). This decrease is likely due to the neuromuscular inhibition regime and the release of active force production as the muscle reaches its eccentric limit. Technological advancements have increasingly utilized computer-based video analysis methods in sports. Notably, a smartphone application (charge a fee) has been developed to calculate the NHEbpa (Soga et al., 2023). This analysis aimed to investigate the effects of the NHEbpa on sprint, COD, and CMJ performance in football players.

The inclusion of NHE-based assessments at critical points throughout the season, for example, by determining players’ strength levels in pre-season to help identify potential deficiencies whilst assessing whether adequate neuromuscular recovery has been achieved prior to return to competition during the recovery phases, provides a cost-effective and practical method for assessing eccentric muscle strength (Opar et al., 2021). Adjusting the intensity of NHE based on the player’s workload—including factors such as match frequency, training intensity, and total weekly training duration—can help maintain muscle balance. However, excessive adjustments in NHE volume are crucial to avoid, as they may lead to muscle soreness. Therefore, it is recommended that a consistent yet minimally effective dose of NHE be implemented (Behan et al., 2023; Cadu, Goreau & Lacourpaille, 2022). Additionally, using a gradual progression strategy to minimize the occurrence of high-intensity matches or training sessions during low-intensity periods and to support eccentric strength development can effectively help prevent hamstring injuries throughout the season (Burgos-Jara et al., 2023; Mendiguchia et al., 2015; Murakami et al., 2023). A meta-analysis examining the effects of NHE on sprint performance and eccentric knee flexor strength indicates that NHE positively influences sprint performance in team sport athletes (Bautista et al., 2021). However, the effects of the NHE and the associated eccentric hamstring strength gains on critical high-intensity movements such as sprinting and COD, vital for sports performance, have not been adequately examined or addressed in the existing literature. This is particularly true for studies measuring improvements in linear speed over distances shorter than 20 m (Ishoi et al., 2018; Mendiguchia et al., 2015). Indeed, despite the ongoing debate regarding the emphasis on COD speed, team sports like football are characterized by multifaceted demands (Girard, Bishop & Racinais, 2013; Scott et al., 2018). Moreover, a hierarchical set of factors influencing COD performance highlights the necessity of investigating whether eccentric exercises, such as the NHE, can enhance COD performance by emphasizing the role of eccentric hamstring strength (Naylor & Greig, 2015).

In addition to these evaluations, recent findings indicate that NHEbpa positively influences sprint performance and strongly impacts COD performance. Specifically, NHEbpa has shown strong correlations with 10 m (r = 0.355, β = 0.007), 20 m (r = 0.444, β = 0.007), and 30 m (r = 0.368, β = 0.014) sprint times, as well as COD performance (r = 0.337, β = 0.026). These results suggest that increased NHEbpa is associated with improvements in high-intensity movement capabilities. NHE exercises are crucial in enhancing COD performance in sports requiring various motor skills, such as football (Moran et al., 2022; Suarez-Arrones et al., 2019). A study investigating an 8-week low-volume NHE intervention in elite football players showed significant changes in linear sprint speed and muscle architecture characteristics with minor effects while producing significant effects on eccentric knee flexor strength and COD performance (Siddle et al., 2024). However, they highlighted a significant improvement in COD performance following the intervention. The effects of NHEbpa on agility are consistent with these findings. However, it is important to note that COD performance is influenced by muscular strength and other factors, including technical skills, reaction time, and coordination (Bustamante-Garrido et al., 2023). The validity and reliability of agility tests that involve repeated changes of direction have been well-established in senior soccer players, reinforcing the importance of such multidimensional factors in COD performance (Brahim et al., 2024). Therefore, while the impact of NHEbpa on COD performance may be more limited compared to sprint performance, this finding underscores the significant role of eccentric strength in the development of COD among football players.

It has been observed that hamstring injuries reported in football generally occur during high-speed running movements (Danielsson et al., 2020). The proposed cause of the injury is attributed to the hamstring muscle’s inability to withstand the forces exerted during its functional demands (high-intensity activities, such as sprinting, rapid deceleration, and COD (Cuthbert et al., 2020). A study by Opar, Williams & Shield (2012) has shown that addressing the architectural features of the biceps femoris (BF) may primarily increase the muscle’s ability to withstand higher extension forces, potentially reducing the risk of injury and improving the muscle’s capacity for controlled shortening velocity during high-intensity activities. Thus, it may reduce the risk of injury due to overextension during eccentric movements (Opar et al., 2021). During sprinting, the long head of the biceps femoris is the hamstring muscle that experiences the greatest elongation (BF ^LH^), which has shorter fascicles compared to the short head of the biceps femoris (BF ^SH^), potentially increasing the susceptibility of the BF ^LH^ to injury (Opar, Williams & Shield, 2012; Thelen et al., 2005).

The potential for increased fascicle length to reduce the risk of hamstring injury and the role of eccentric hamstring strength in mitigating this risk has been noted. Our analysis’s findings indicate that a one-unit reduction in NHEbpa correlates with a decrease of approximately 0.007 s in 10-m and 20-m sprint times and about 0.014 s in 30-m sprint times, suggesting a modest but beneficial enhancement in sprint performance. Although these changes may seem small, they represent incremental improvements in sprint performance and may accumulate throughout a season or over multiple training sessions, producing beneficial effects.

A 6-week NHE intervention program has demonstrated significant improvements in eccentric knee flexor strength and 10-m sprint performance and immediate enhancements in COD speed post-intervention. These improvements were sustained after 3 weeks of no training (Pollard et al., 2019). These findings have important implications for designing training programs and planned rest periods, such as the winter break in European football (Ekstrand, Spreco & Davison, 2019). Additionally, the results suggest that the intensity of contraction in response to eccentric exercise may be a more decisive factor than training volume in triggering adaptive responses in the hamstrings. This suggests that mechanical tension on muscle fibers and neuromuscular stimulation may play a critical role in muscle hypertrophy and strength adaptations. In particular, higher-intensity eccentric contractions may provide greater neurophysiological and structural adaptations by increasing motor unit activation and intramuscular tension. However, further studies will be required to confirm this proposition.

While these findings contribute valuable insights, some limitations must be acknowledged. Participants were instructed to exert maximal resistance during the descent phase of the NHE until ground contact. However, the consistency of the range of motion and speed during the NHE may have varied among participants, potentially affecting architectural adaptations. Despite this variability, the prescribed NHE examines protocol reflecting real-world practice, enhancing the practical relevance of the study’s findings. Previous research has shown that interventions applied with varying degrees of knee extension during the NHE have minimal effects on muscle architecture adaptation (Bloomquist et al., 2013; Guex & Millet, 2013).

## Conclusion

This analysis shows significant relationships between the NHEbpa and sprint times, COD, and CMJ performance. However, it is also important to report the magnitude of these relationships, as the correlations explain only 16–44% of the variance, which can be considered relatively low in practical terms. In particular, it was found that COD performance had a significant effect on NHEbpa with 10, 20 and 30-m sprint times, but CMJ performance did not show a significant effect. Ridge Regression analysis, employed to address the high multicollinearity observed in multiple linear regression, provided more robust and reliable interpretations of the relationships among these variables. These findings emphasize the critical role of sprint and COD training in improving NHE performance in football players. However, the extent to which this relationship is applicable in practice and meaningful performance increase should be evaluated with statistical significance, real game dynamics, and individual performance gains of the athletes. In addition, further investigation of different eccentric exercise interventions is needed to understand better the dose-response relationship between eccentric strength and motor performance characteristics of the hamstring muscle group. However, such studies must be designed considering the limitations related to the consistency of the NHE protocol.

### Limitations

While these findings contribute valuable insights, some limitations must be acknowledged. First, although the relationships between the NHEbpa and sprint, COD, and CMJ performance were statistically significant, the explained variance of the correlation coefficients ranged between 16% and 44%. This indicates that while the relationships are significant, their effect sizes are relatively small. Therefore, the findings should be interpreted more cautiously regarding practical significance. For example, although the regression coefficients in Tables 3 and 6 show small effect sizes, the study still substantially contributes to the literature. The findings suggest that NHEbpa influences sprint, COD, and CMJ performance, providing an important foundation for future research. Secondly, participants were instructed to exert maximal resistance during the descent phase of the NHE until ground contact. However, the consistency of the range of motion and speed during the NHE may have varied among participants, potentially affecting architectural adaptations. Despite this variability, the prescribed NHE examination protocol reflects real-world practice, enhancing the practical relevance of the study’s findings. Previous research has shown that interventions applied with varying degrees of knee extension during the NHE have minimal effects on muscle architecture adaptation (Bloomquist et al., 2013; Guex & Millet, 2013).

This study highlights the distinction between statistical and practical significance, emphasizing that studies with larger samples and different populations could more strongly support the results.

### Practical applications

Coaches obtain vital information about sprint and COD performance by understanding the effects of NHEbpa. Players can expect better performance in high-intensity movements when NHEbpa levels rise. Coaches gain the ability to design personal training approaches through systematic NHEbpa-level monitoring of their athletes. Performance enhancement and injury prevention success can be achieved through targeted eccentric hamstring strength training interventions aimed at NHEbpa. The assessment of NHEbpa at scheduled intervals brings value to training development.

## Supplemental Information

10.7717/peerj.19275/supp-1 Supplemental Information 1 Raw Data.

## Additional Information and Declarations

### Data Availability

The following information was supplied regarding data availability:

The raw data is available in the Supplemental File.
